# Gesture Recognition Framework for Teleoperation of Infrared (IR) Consumer Devices Using a Novel pFMG Soft Armband

**DOI:** 10.3390/s24186124

**Published:** 2024-09-22

**Authors:** Sam Young, Hao Zhou, Gursel Alici

**Affiliations:** Applied Mechatronics and Biomedical Engineering Research (AMBER) Group, School of Mechanical, Materials, Mechatronic and Biomedical Engineering, Faculty of Engineering and Information Sciences, University of Wollongong, Wollongong, NSW 2522, Australia; samalexyoung@outlook.com (S.Y.); hzhou@uow.edu.au (H.Z.)

**Keywords:** gesture recognition, teleoperation, force myography, pneumatic myography, armband, machine learning, infrared

## Abstract

Wearable technologies represent a significant advancement in facilitating communication between humans and machines. Powered by artificial intelligence (AI), human gestures detected by wearable sensors can provide people with seamless interaction with physical, digital, and mixed environments. In this paper, the foundations of a gesture-recognition framework for the teleoperation of infrared consumer electronics are established. This framework is based on force myography data of the upper forearm, acquired from a prototype novel soft pressure-based force myography (pFMG) armband. Here, the sub-processes of the framework are detailed, including the acquisition of infrared and force myography data; pre-processing; feature construction/selection; classifier selection; post-processing; and interfacing/actuation. The gesture recognition system is evaluated using 12 subjects’ force myography data obtained whilst performing five classes of gestures. Our results demonstrate an inter-session and inter-trial gesture average recognition accuracy of approximately 92.2% and 88.9%, respectively. The gesture recognition framework was successfully able to teleoperate several infrared consumer electronics as a wearable, safe and affordable human–machine interface system. The contribution of this study centres around proposing and demonstrating a user-centred design methodology to allow direct human–machine interaction and interface for applications where humans and devices are in the same loop or coexist, as typified between users and infrared-communicating devices in this study.

## 1. Introduction

The modern household in Australia contains, on average, over 19 connected devices and is forecasted to have more than 30 within the decade [1], many of which have a means of interaction/communication with users. These devices are diverse in their range of purpose, functionality, ergonomic attributes and technological advancement. Similarly, the users of these devices are diverse in their communication methods, physical and cognitive abilities, desired intents and technological capabilities. Considering the amount of existing technology and the variance in their production methods, it has become evident that a backward functionality solution is needed—a solution that can map the unique needs of the individual to the unique functions of existing devices.

Current research trends show a growing interest in utilising gesture recognition as a means of human–machine interaction, redefining the concept of user interfaces with consumer electronics of different sizes and capabilities [2]. Gestures are expressive, purposeful body motions that utilise the physical manipulation of a person’s hands, arms, face and body to interact with their surrounding environment or to communicate information. Gesture recognition has thus become highly regarded as a reasonable and efficient means of human–computer interfacing [3].

Hand/arm gesture recognition is utilised in a diverse range of topics including prosthesis control, human–computer interaction systems, sign language recognition, musical creation, robotics control and entertainment/virtual reality interfaces [4,5,6,7,8]. The methods employed to recognise gestures include motion tracking, computer vision, electromyography (EMG) [9], force myography (FMG) [10,11] and mechanomyography (MMG) [12]. This work uses a custom-built pressure-based force myography (pFMG) method to acquire user intent [13].

Although optical tracking systems, both marker-based and markerless, are often preferred over EMG and FMG for gesture recognition due to their precision and robustness, these systems have notable drawbacks that make them less practical for everyday use. Marker-based tracking requires the placement of physical markers on the user’s body, which can be cumbersome, intrusive and uncomfortable, especially for prolonged use. Markerless systems often suffer from occlusion issues with user’s gestures blocked from the camera’s view, leading to unreliable recognition. Additionally, markerless systems can be computationally intensive, requiring substantial processing power and sophisticated algorithms to achieve real-time performance, which can be a barrier to widespread adoption.

In contrast, pFMG can offer a more practical and user-friendly approach to gesture recognition, suitable for real-world use. The pFMG sensors used in this paper can detect changes in muscle pressure, providing a direct and reliable measure of muscle activity without the need for visual tracking. This eliminates the occlusion problems inherent in optical systems and reduces the computational load, facilitating faster and more efficient real-time processing. Furthermore, pFMG sensors are non-intrusive and can be comfortably worn for extended periods, making them suitable for continuous use.

This body of work is an extension and application of our previous work developing a soft pressure-based force myography armband [13]. In the previous research, a novel non-invasive, lightweight and low-cost wearable armband was developed for human–machine interface applications. This soft pFMG armband uses air pressure sensors to capture mechanical biosignals from muscle activity, providing stable, reliable, and reproducible data with minimal calibration. The armband’s design ensures durability and resistance to external factors like sweat and mechanical deformation. This work extends its capabilities by providing the “intelligence” to the armband—adding a gesture recognition framework and teleoperation control capabilities.

To demonstrate the practical impact and provide a foundation for future research, empirical evidence of the proposed system’s real-world relevance and market potential is presented by integrating teleoperation capabilities for infrared (IR) communicating devices. By focusing on IR, it is highlighted how the proposed system can enhance user experience and interaction with everyday devices. The abundance of IR communicating consumer electronics with its wide range of functionality achievable across devices makes it an excellent medium for control. IR ports are common in televisions, DVD players, sound systems, air conditioners, drones, robots, toys and many other household peripherals. IR technology uses the infrared component of the electromagnetic spectrum (870–950 nm), requires a direct line of sight and cannot pass through walls, which can provide a level of privacy and security not achievable by other wireless communication protocols [14]. These factors make IR an effective protocol for the purposes of this study, though future work could benefit from incorporating additional wireless communication methods to supplement the range of targetable devices and leverage their transmission features.

Furthermore, IR devices have been common in households for many years, and are familiar/intuitive to control for all age groups. The combination of multiple devices being controlled by different remotes with numerous buttons can result in households with an overwhelming amount of functionality [15]. With this considered, it is easy to understand how users with cognitive or physical impairments can be disadvantaged when navigating these controls. Ergonomics studies reveal that the majority of users only engage with the direct action controls of their remote devices (i.e., power, play, volume, channel buttons, etc.) [16]. A study [17] reported that remote control usability is not only defined by the traditional work-related definition but also “emotional usability”, which includes additional needs such as enjoyment and entertainment which enhance the users’ product experience.

Our work integrates the benefits above of pFMG and IR technology with a user-centred design methodology to enable direct human–machine interaction with infrared-communicating devices through a novel, pressure-based soft robotic armband. This armband captures user intent and teleoperates targeted IR devices by recognising five different hand gestures (sufficient for action controls) using machine learning algorithms. The key contributions of this paper are:

- This is the first study to investigate and demonstrate the feasibility of using an FMG-based gesture recognition system for teleoperating consumer IR devices.
- Two implementations of the gesture recognition system, the original framework and the extended framework, are proposed and implemented. All phases of the system architecture are thoroughly detailed and analyzed, resulting in a modular solution that adapts to individual users and various devices. Additionally, it is designed to be sufficiently fast, enabling ‘plug and play’ capabilities.
- The selection of feature sets and classifiers are comprehensively studied to identify the optimal configurations for pFMG-based hand gesture recognition.
- Our proposed system demonstrates a state-of-the-art performance in both recognition accuracy and prediction speed when compared to the existing literature incorporating FMG- or EMG-based gesture recognition systems with similar gesture classes.
- This research provides empirical evidence of the system’s practical impact and market potential, emphasising its real-world relevance and facilitating reproducibility for further optimisation. The scientific contribution of this work is especially beneficial to people with upper limb loss/deficiency, allowing them to teleoperate household devices without the need for an intermediary prosthetic limb. The device design and recognition implementation do not require specific armband orientation or placement, as it maps overall muscle intent rather than targeting specific muscles.

## 2. Materials and Methods

This section presents the equipment, the proposed original framework and extended framework methods to teleoperate various IR consumer devices by hand gestures. The main equipment includes a custom-built pFMG armband and an IR emulator device. In the original framework, all the steps are described in detail, including IR and pFMG data acquisition, pre-processing, feature construction and selection, classifier model selection, gesture mapping, post-processing, and device actuation. After adding a motion sensor (IMU), an extended framework method is proposed to further enhance the functionality of the system with spatial augmentation, quasi-dynamic gestures, and proportional control of a linked device.

### 2.1. Equipment

**Figure 1:**
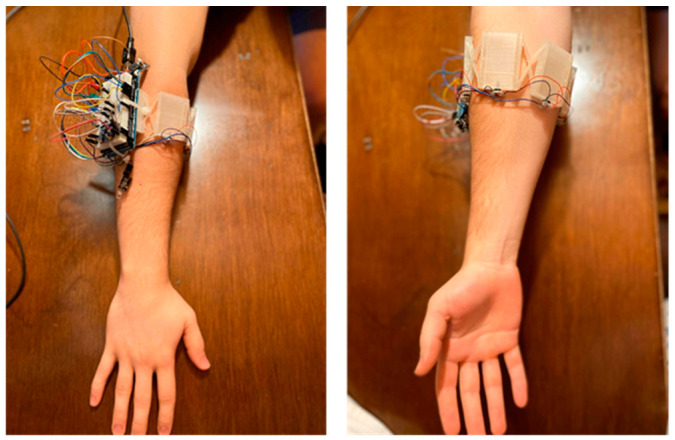
Novel pneumatic myography (PMG) armband [13].

A novel pFMG armband prototype (shown in Figure 1) is developed for FMG data acquisition. The armband is made up of five 3D-printed pressure-sensitive chambers (PSCs) that are soft and flexible using Ninjaflex as one of the commercially available flexible filaments. Physical expansion or contraction of the forearm muscles during a gesture execution deforms the chamber which in turn generates internal pressure changes that can be detected by the attached amplified pressure sensors (Honeywell ABPDANT015PGAA5). Our previous work [13] demonstrated that five chambers were sufficient to fully encompass the forearm tightly whilst keeping desirable design features such as modularity (add/subtract sensing chambers) and scalability (increase/decrease diameter) for individual users.

The mechanical characteristics of the Pressure Sensitive Chambers (PSCs) were proven to be linear, stable over time, show insignificant hysteresis and attain a high lifetime cycle (greater than 1.5 million cycles to failure). The PSC’s insensitivity to drift means that there is minimal need for re-calibration, which is a primary advantage of this pressure sensing system over existing piezoresistive force/strain sensors on the market. The armband serves as a low-cost (approximately AUD 129 for all the materials) and effective alternative to traditional EMG and FMG sensors. The armband is user-friendly, fully customisable and stretchable, increasing comfortability as it can be worn over clothes without performance effects occurring. The comprehensive details of the armband are introduced in [13].

A low-cost infrared emulator device is developed by using off-the-shelf components and incorporated with the pFMG armband using an Arduino Mega2560 (Arduino LLC, Turin, Italy). An infrared receiver module was placed towards the back of the device to record incoming infrared codes transmitted from existing remotes. An infrared transmitter module was placed at the front of the device to transmit the stored infrared codes back to the peripheral devices when a gesture is performed. A 9-DOF (degrees of freedom) IMU was placed centrally forward on the remote and has capabilities to capture 9 distinct types of orientation- or motion-related data: 3 DOF each of angular velocity, acceleration and magnetic orientation.

An infrared library by Ken Shirriff [18] was used for decoding and transmitting the received IR signals. Finally, machine learning tools from the Scikit-learn software package [19] in Python (v3.7.1) were used. All processing was performed on a laptop with Intel^®^ Core™ i7-8550U CPU (Intel, Santa Clara, CA, USA) and 8GB of installed RAM. It is recognised that this is a prototype design and there are easily a few components that can be replaced with smaller, more efficient and/or customised alternatives. The final device, including the armband and the IR module, weighs approximately 124 g and costs approximately AUD 210 in its prototype form.

### 2.2. Framework Method

The system framework has been broken down into distinct phases, both in terms of reporting and the system architecture. These phases are as follows:

- Infrared data acquisition—Infrared signals are recorded from their target devices and stored for future emulation.
- pFMG data acquisition—Raw force myography signals are recorded from the forearm muscles of the user when performing gestures.
- Pre-processing—The raw pFMG data are filtered and rectified to establish a form that is suitable for featurisation and ultimately be fed into a classifier model.
- Feature construction and selection—The processed data are formatted and fed into mathematical operations that help differentiate and diverge gesture data.
- Classifier model selection—Classifier models learn how to predict gestures based on training and test datasets from the featured data.
- Gesture mapping—Gestures are mapped to infrared device functionalities.
- Post-processing—Predictions from classifier models are collected and refined to improve the stability and accuracy of final predictions.
- Device actuation—Final predictions are used to control devices according to gesture mapping setup.

**Figure 2:**
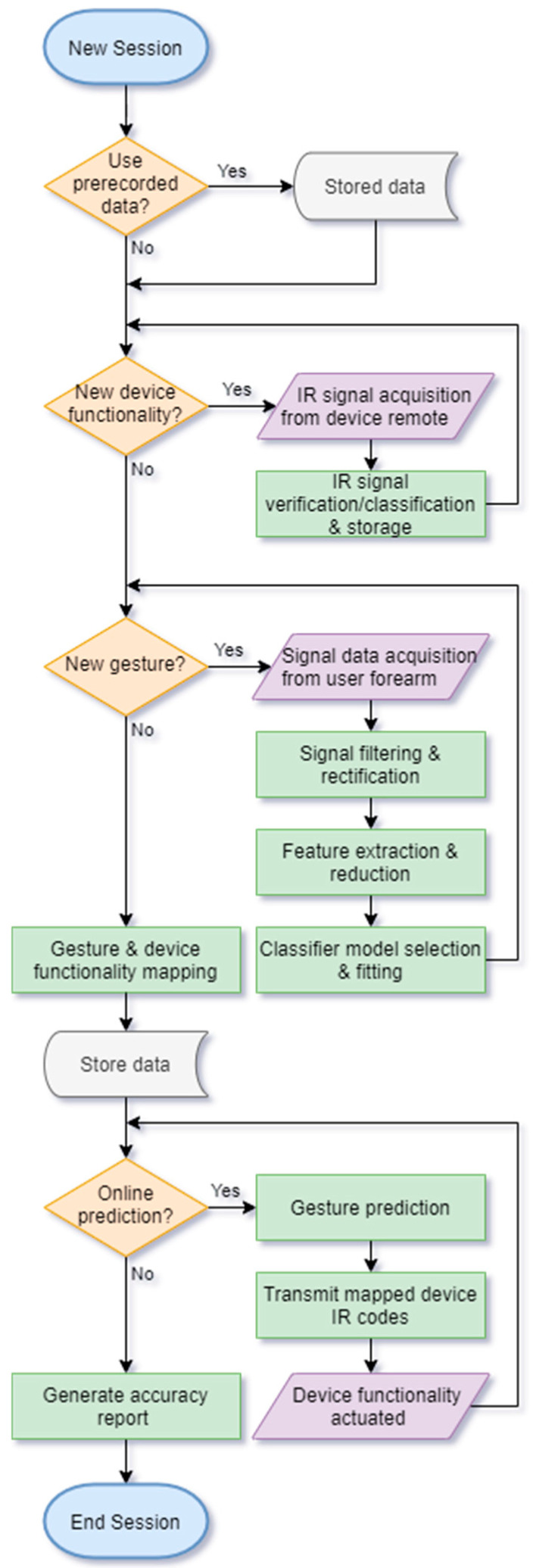
Gesture recognition and device teleoperation framework.

The benefit of this modular nature is that entire system capabilities can be modified relatively easily whilst maintaining a functioning workflow. Figure 2 presents a simplified overview of the framework process in the form of a visual process flowchart.

#### 2.2.1. Infrared Data Acquisition

IR codes were recorded by actuating desired buttons of existing IR remotes towards the direction of the infrared receiver. A direct line of sight was maintained to ensure the IR codes were received correctly, i.e., not intercepted, scattered or reflected by different mediums and surfaces. An Arduino script, together with a popular IR remote library by Ken Shirriff [18] decoded the incoming signals based on the protocol pattern received. Our framework has accounted for 17 unique manufacturer protocols and their sub-variations that incorporate a large and varied pool of IR consumer goods (common protocols identified include NEC, Sony and RC5). To account for codes that are not predefined as a particular manufacturer type, our framework simply records the raw code data. This data information is in the form of pulse bursts and space timing ticks which can be stored and replicated in its raw form for future use. This method could have been used for all received IR signals but the former method allows for optimisation and easier identification.

#### 2.2.2. pFMG Data Acquisition

**Figure 3:**
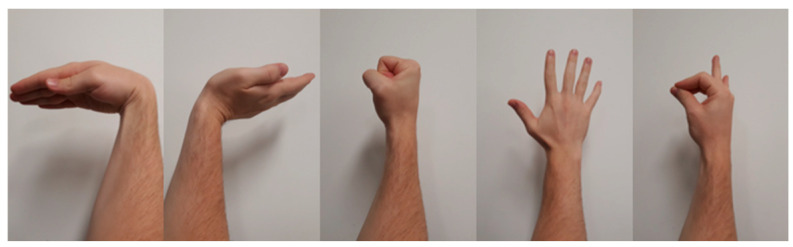
Gestures performed by subjects—(left to right) Wave In, Wave Out, Fist, Spread Fingers and Pinch.

As shown in Figure 3, five common gestures (Fist, Pinch, Spread Fingers, Wave In and Wave Out) were selected to test the effectiveness of the gesture recognition system. These gestures were selected for a number of reasons, the main one being that they are relatively prevalent in the myography-based gesture recognition literature. These gestures are frequently cited as possessing the following desirable characteristics [20,21,22]:

1. They are discernible enough to be detected by most types of sensors, EMG, FMG, MMG, etc.
2. They are distinguished from each other, i.e., use differing muscle groups during actuation and are sufficiently different from resting position.
3. They are simple to understand and perform. It is recognised that patients with arm/hand disabilities/amputations may still find it difficult or impossible to perform the gestures; however, user intention is relatively clear when performing these gestures (which can still be detected by myography-based systems).
4. They are comfortable to perform, i.e., reduce induced muscle pain and fatigue.
5. They are intuitive to the user in performing operations and/or actuating devices.

It should be noted, however, that a large body of the published literature utilises EMG-based acquisition devices and thus does not truly reflect the optimal gestures that could be used for the FMG-based method utilised in this study. Finding the optimal gestures for FMG applications was deemed beyond the scope of this study and the disadvantages of using EMG-refined gestures were outweighed by the need for the literature comparability.

For the actual implementation of gesture data acquisition, the pFMG armband was placed over the belly of the forearm (slightly distal to the elbow). The orientation of the armband was adjusted to ensure the base was situated slightly medial to the anconeus muscle. The base contains no pressure chambers and was placed on this region as it has little muscle deformation relative to other sites. Proper orientation was not crucial for gesture recognition but the same orientation between sessions was required unless a user chose to define new gestures or redefine existing ones.

Raw FMG data were recorded by the pFMG armband as a stream of arrays at a sampling period of 1.35 ms (740 Hz), each containing five elements (for each pneumatic chamber). For training the machine learning algorithms, a single gesture recording was defined as obtaining 2000 pFMG data samples which equated to approximately 2.7 s of data. This amount of data was deemed sufficiently long to encompass a static pose whilst not causing fatigue effects during training sessions when the user would have to repeat gestures multiple times in a row. The number of times a recording trial (each of the 5 gestures performed for 2.7 s) was repeated was set to 3. Utilising 3 separate recordings of a 3-second gesture (with other gestures interspersed) instead of a single 9-second gesture recording increased the variability in subject data, preventing the likelihood of overfitting and reducing muscle fatigue. Users invariably made discrete adjustments to their actuation of a gesture upon re-initiation.

#### 2.2.3. Pre-Processing

**Figure 4:**
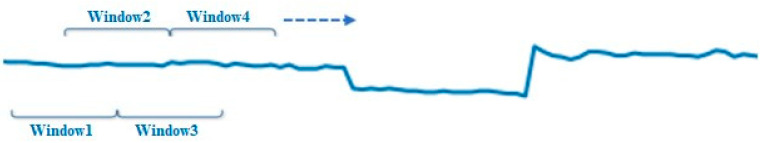
Sliding window implementation with 50% overlap (not to scale).

In preparation for featurisation, the gesture data were segmented by defining a ‘window’ of 100 samples (approximately 135 ms worth of data) and stepping through the data with a stride length of 50 samples (allowing for 50% overlap) as depicted in Figure 4. Sliding window implementation with 50% overlap (not to scale). For a given gesture recording length, this would produce (N/S − 1) windows, where N is the number of samples and S is the stride length. Due to the consistent, steady nature of the pressure and orientation readings, it was identified that little rectification or smoothening was required prior to featurisation. As a precautionary measure, a 5th-order digital bandpass filter was utilised to limit a 10% sampling rate margin of error. This would ensure that any lag or lead effects would be disregarded and alert the user of invalid data. No such alerts were recorded during the process of experimentation.

#### 2.2.4. Feature Construction and Selection

Featurisation is the process of transforming raw FMG signals obtained from the armband into features that better represent the data to classifier models. This increases the predictive capabilities of the machine learning models and improves their accuracy when subjected to unseen data. In this study, this featurisation is achieved through the application of mathematical operations to each window of gesture data. A total of 17 time-domain features were initially applied including Integrated Absolute Value (IAV), Waveform Length (WL), Average Amplitude Change (AAC), Slope Sign Change (SSC), Simple Square Integral (SSI), Root Mean Square (RMS), Mean Absolute Value (MAV), Modified Mean Absolute Value Type 1 (MAV1), Modified Mean Absolute Value Type 2 (MAV2), Zero Crossing (ZC), 3rd Temporal Moment (TM3), 4th Temporal Moment (TM4), 5th Temporal Moment (TM5), Willison Amplitude (WAMP), Difference Absolute Standard Deviation Value (DASDV) and Myopulse Percentage Rate (MYOP) (see [23,24,25,26,27,28] for mathematical descriptions and operations). The final list of the features selected for the purpose of this study is presented in Section 3.4.

Feature construction transforms a single window of gesture data into a ‘featured window’ which is a comprehensive mathematical description of approximately 135 ms of data from a single sensor. A ‘featured frame’ is an amalgamation of each sensor’s featured windows. Therefore, a single gesture can be effectively defined as an array of frames. Intra-gesture variation in features was determined by subjecting element-wise relative standard deviation (coefficient of variation) tests. Inter-gesture variation in features was obtained by applying coefficient of variation tests on the intra-gesture means (mean values between the frames of all compiled gestures).

Feature selection was based on the results of the intra-gesture and inter-gesture variances. A feature was considered highly effective if it was characterised by both low intra-gesture variability and high inter-gesture variability. This demonstrated that the gesture was consistent during its execution and/or between trials but sufficiently different from other defined gestures to be easily distinguished. This method of feature selection follows a filter-based approach, that is, it chooses the optimal features without involving the final classifiers in the process (as is the case for wrapper-based methods). This method prevents the influence of overfitting errors affecting feature performance. Furthermore, a filter-based approach has a significant reduction in processing time when compared to wrapper-based approaches because the latter must iterate the selection process with every combination of feature and classifier model (an exponential function). Following feature selection, the data are formatted into a dimensionality that is suitable for feeding into the classifier models.

#### 2.2.5. Classifier Model Selection

Similar to the protocol used in our previous work [29], the test pool of classifier models started with Decision Tree, Random Forest, k-Nearest Neighbour, Linear Discriminant Analysis (LDA), Quadratic Discriminant Analysis (QDA), Nu-Support Vector Classification (NuSVC), and Gaussian Naïve Bayes (refer to [30,31] for further details). These classifier models are well developed in the existing literature and have substantiative documentation for their implementation.

For model evaluation purposes, the trial datasets were split into training and testing sets by the ratio 2:1. Therefore, for every three trials recorded, one trial was hidden from the classifier for future testing purposes. Every unique combination of hidden trials was used to determine the overall classifier accuracy for the given data set. Therefore, the overall classifier accuracy is based on the average of the binomial coefficient accuracy scores according to the binomial coefficient equation:

(1) C(n,r)=n!/(r!(n−r)!),for r trials from a set of n trials

As a total of 6 trials were recorded per user in the experimental procedure, the classifier accuracy was taken as the average of 15 unique accuracy scores. This method of estimating the skill of the classifier models is essentially a customised version of a k-fold cross-validation method. The primary difference between the two is that due to the relatively small data collection size and the use of static gestures, complete segregation of the trials was necessary to avoid overfitting.

#### 2.2.6. Post-Processing

Due to the high-frequency sampling rate and small data window sizes, the classifiers were able to produce approximately 10 gesture predictions per second. Post-processing was performed on the system to condense these predictions into a single prediction which increases the stability and accuracy of the results. A prediction queue was established and after it was filled to a maximum of 10 predictions, the mode value was taken as the final prediction. Note that if the mode value did not constitute at least 75% of the queue, the predictions were deemed too random, the gesture was labelled as ‘Unknown’ and no IR codes were transmitted. If desired, the last confident prediction can be used as the current gesture during these ‘Unknown’ gesture periods. This is useful for scenarios where the user is experiencing fatigue as a result of transmitting repeat codes over an extended period of time.

#### 2.2.7. Gesture Mapping to IR Codes

After both gestures and IR codes were recorded, the user was given the option to map gestures to any number of IR codes. Assigning the same gesture to multiple codes allows for the teleoperation of multiple devices using the same gesture. This is beneficial when a gesture is intuitive for multiple cases, for example, an outwards wave gesture could allow for both turning up the volume on a speaker and temperature on an air-conditioner. Mapping multiple IR codes to the same gesture also allows for synchronous teleoperation of multiple devices. This technique is, however, limited by the line-of-sight transmission angle that is a characteristic of infrared communication. Alternatively, the user could map multiple gestures to the same infrared code, though this would only serve to provide added redundancy in the system as a backup in the case of poor gesture recognition.

#### 2.2.8. Device Interfacing and Actuation

The current framework’s device interfacing capabilities were tested on a variety of infrared communicating devices. To demonstrate the efficacy of the system, a number of factors were taken into consideration when selecting devices to actuate:

1. The devices must be prevalent in society, i.e., commonly used by people in a variety of environments including homes, offices and factories, etc.
2. The devices must demonstrate a variety of consumer electronics, i.e., showcase actuation of television units, air-conditioning units, sound systems and video players, etc.
3. The devices should utilise a variety of manufacturer protocol codes with different modulation methods, i.e., NEC, RC5, Sony and Mitsubishi, etc. using 12- and 16-bit protocols.

IR codes were recorded onto the IR transceiver by triggering commands from IR actuators (e.g., TV remote). These recorded codes (unique to a given manufacturer, device and button) were subsequently transmitted back to the controlled end device (e.g., TV) when the mapped gesture had been activated. Note that IR codes were only transmitted when a gesture was changing state, that is, repeat recognition of the same gesture did not result in repeat transmission of the IR codes as this prevented debouncing-characteristic behaviour. The current gesture and IR code transmissions were observed by the user in real-time in text form within the application package.

### 2.3. Extended Framework Method

#### 2.3.1. Spatial Augmentation

The extended framework follows the same general phases as the original framework (Section 2.2) with the exception of spatial augmentation added to the actuation capabilities. This was primarily carried out as a proof of concept with the goal of achieving quasi-dynamic gesture recognition functionality. This framework was intended to be an extension and thus the aim was not to alter the foundation of the original framework. This would mean that the augmentation capabilities could be turned on and off seamlessly in real-time as desired without affecting performance.

Spatial data were added as part of the extended framework by incorporating a 9-DOF motion sensor, IMU, (BNO055, Bosch Sensortec, Reutlingen, Germany) in the pFMG armband. Although the sensor had an in-built accelerometer, gyroscope and magnetometer, these features were omitted as the framework was only concerned with the absolute orientation of the forearm. Modules of the framework do allow for future expansion, incorporating information such as angular velocity and acceleration vectors. Acceleration and velocity data acquisition was found to require a more rigorous calibration setup, sometimes taking as long as 30 s to ensure a sufficiently high confidence level. The nature of this framework was to promote fast and easy utilisation by end users and so it was decided to neglect this information for this version of work and utilise Euler vectors as a means of spatial augmentation. Sampled at a rate consistent with the original framework (1.35 ms), an Euler vector provides absolute orientation information in three axes (x, y, and z).

Initially, the orientation data were included within the featurisation process; however, this immediately presented a significant issue. Defining gestures with integrated orientation values meant that every new orientation was defined as a different gesture. This made the method impractical as there were infinite orientations in 3D space. A potential fix would be to place a sensor onto the torso of the user to give an initial frame of reference and allow for relative orientation data. This, however, poses issues in itself; the extra sensor increases the bulkiness of the final devices, requires an attachment mechanism, increases the overall cost, and technical complexity and furthermore the user’s torso would have to remain still.

The alternative solution was to remove the orientation data from within the feature and classification process and instead use it within the post-processing phase of the framework. This meant that orientation data were only considered in a complementary form after a gesture had been predicted. This had three primary benefits:

1. As a complementary approach, no significant modifications to the original framework were necessary.
2. Spatial gestures were now relative to the starting position of the gesture (as opposed to the infinite starting positions relative to the 3D orientation space).
3. Gestures were now more stable across different orientations. For example, prior to spatial augmentation, slight muscle deviations caused by changing the orientation while holding a static gesture may have influenced the prediction of a gesture. With spatial augmentation, the gestures were more ‘locked in’—i.e., it was recognised that the user had moved whilst holding a gesture (less likely to be perceived as a new gesture altogether).

#### 2.3.2. Quasi-Dynamic Gestures

**Figure 5:**
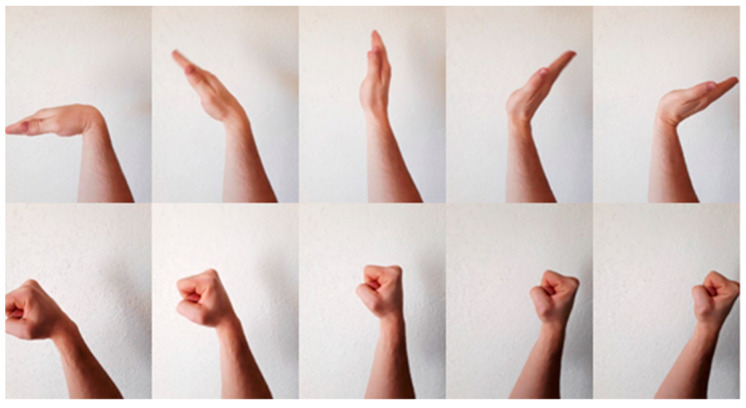
Comparison between dynamic gestures (**Top row**) and quasi-dynamic gestures (**Bottom row**).

Spatial augmentation allows for quasi-dynamic gestures to be recognised in the extended framework. A quasi-dynamic gesture differs from a dynamic gesture in that the former actuates a constant gesture (no transient motion) with variable orientation and the latter actuates a transformative gesture (with transient motion). There are a few reasons as to why quasi-dynamic gestures were implemented instead of dynamic gestures in this study. One of the key components of this system is its ability to perform FMG data acquisition, pre-processing, featurisation and classification prediction in a matter of milliseconds (approximately 10 predictions can be made per second). This speed is possible as a result of the real-time sliding window implementation with small packets of data, essentially taking many snapshots of the data to understand discrepancies in motion. This implementation is not as well suited to dynamic control as it is for quasi-dynamic control. Dynamic control requires a lagging window and holding off on final predictions to ensure a gesture is completed first. Without any modifications, the original framework would potentially identify the dynamic wave gesture in the top row of Figure 5 as a series of discrete gestures, quickly transitioning between Wave In, Rest and Wave Out (with perhaps ‘Unknown’ in-between states). Another consideration of dynamic gestures would be the temporal parameter of movement speed. There are virtually an infinite amount of speeds that the gesture could be actuated with and thus the movement will have to be scaled in real-time (with dynamic time warping algorithms) to measure similarities between the trained and recorded temporal sequences.

Quasi-dynamic gestures do not require any scaling as the gesture relative to the armband is constant. This significantly reduces the processing power required and processing time until a final prediction as no lagging is necessary. The quasi-dynamic gesture in the bottom row of Figure 5 is still considered to be a static Fist gesture in the eyes of the classifiers but the orientation change allows for additional functionalities to be added during the post-processing stage.

#### 2.3.3. Proportional Control

Proportional control is a linear feedback control system in which a correction is applied to a given variable with a magnitude that is proportional to the difference between a desired and recorded value. In this extended framework, proportional control was possible by changing the orientation of the arm whilst a gesture was being held. The setpoint orientation was established during the initiation of the gesture and subsequent movements determined the process variable. The difference between the instantaneous orientation values of the initial and current poses influenced the magnitude of the controller output in a linear fashion. Depending on the gesture being performed, only one axis was considered (though multiple axes could be considered independently). To clarify with an example, a ‘Fist’ gesture that controls an LED strip was activated in an anterior orientation before the arm was laterally rotated. In this example, the system was designed to compute the difference in the orientation of the longitudinal (craniocaudal) axis. The magnitude of lateral rotation from the starting setpoint was then subsequently used to set the brightness of the LED strip. As the infrared codes were transmitted in discrete packets as opposed to a continuous stream, the proportional control was used to either determine the rate of sending the same codes (e.g., increase television volume faster when laterally rotated) or incrementally select the next stored infrared code (increase television channel number when laterally rotated).

#### 2.3.4. Extended Framework Gesture Dataset

To the best of our knowledge, there are no established gesture datasets for the accuracy comparison of quasi-dynamic gesture recognition systems. This study therefore determined to test the augmented version of the five common gestures used in the original framework. These quasi-dynamic gestures were as follows: Fist with supination/pronation rotation; Pinch with medial/lateral rotation; Spread Fingers with arm flexion/extension; Wave In with flexion/extension and Wave Out with medial/lateral rotation. These covered rotations about the longitudinal (craniocaudal), anteroposterior (dorsoventral) and horizontal frontal (mediolateral) axes.

Using the same procedure as the original framework, the raw FMG data were recorded from the upper forearm by the pFMG armband at a sampling period of 1.35 ms. In total, 2000 FMG samples (2.7 s of data) were obtained per gesture for a single trial with 3 trials recorded in total for a user session. The only adjustment was that users were asked to perform the orientation motions slowly during the 2.7 s recording time, taking care to keep the gesture consistent and ensure the pressure chambers were not pressed against the body during rotation.

## 3. Results

This section presents the hand gesture recognition performance using the pFMG armband and the proposed framework methods. Some better-performing features and classifiers are identified. The offline classification results show comparable gesture recognition accuracy to some previously reported work. Furthermore, the feasibility of online teleoperation of various IR devices by the proposed system is successfully demonstrated. 

### 3.1. Participant Subjects

The gesture recognition accuracy component of the original framework was evaluated by utilising 12 able-bodied subjects (9 males, 3 females, age 23.1 age 23.1 ± 2.2 years; mean ± SD). The experiment methodology was approved by the Human Medical Research Ethics Committee at the University of Wollongong and each participant signed consent forms for their data to be included within this report. The inclusion and exclusion criteria for participant recruitment a described below.

Participants must meet the following:

- Be 18 years of age or older.
- Have no known musculoskeletal or neurological disorders affecting the upper limbs.
- Be able to understand and follow instructions in English.

Participants will be excluded from this study if they meet the following:

- Have skin conditions such as dermatitis or hypersensitivity that could be aggravated by contact with electrodes or wearable devices.
- Have epilepsy or a history of seizures, as the experiment may involve visual or auditory stimuli that could trigger an episode.
- Are pregnant, as the effects of some sensory modalities on pregnancy are not well studied.
- Have recently undergone surgery on their hands, wrists, or forearms.

pFMG data from the forearm muscles of their dominant hand were recorded using the pFMG armband. Each participant was asked to wear the pFMG armband on the belly of their dominant hand’s forearm, positioning the base chamber (which contains no sensors) slightly medial to the anconeus. If the armband was deemed to have insufficient contact pressure (perhaps due to a smaller forearm circumference), then the base chamber was detached to reduce the armband circumference. For the offline analysis of gesture recognition using the original framework, 5-channel pFMG data were recorded in two sessions for each participant without any reported effects of fatigue. In each session, a single trial consisted of performing the five gestures (Wave In, Wave Out, Spread Fingers, Fist and Pinch) in a random sequence for approximately 3 s each. The trial would then be repeated twice more for a total of three trials per session, each gesture containing approximately 9 s worth of data. The entire session would then be repeated, resulting in 18 s worth of data per gesture.

For the extended framework, only 3 participant subjects (1 male, 2 females, age 32.3 ± 13.2 years; mean ± SD) were available for testing. This was deemed insufficient for robust conclusive results but proceeded nonetheless as a proof of concept and for experimentation purposes.

### 3.2. Infrared Emulator Results

The infrared codes of all devices tested were able to be received, stored, emulated and transmitted by the infrared emulator device. It was noted that manufacturer codes differ in the number of bits used (e.g., NEC uses 32 bits while Sony uses 12 or 20 bits). Some manufacturer codes are sent numerous times for each button press and a few remotes were noted to use more than one type of manufacturer code. Additionally, holding down a button transmits repeated codes for some remotes whilst for others it produces a different, symbolic code altogether. The current method of overcoming these issues is to manually specify the above parameters.

While all tested devices were eventually able to be replicated, the most challenging emulations stemmed from unknown manufacturers where the protocol modulation scheme is indeterminate. For these cases, the codes were recorded in their raw form and labelled as ‘Unknown’ type. This was recorded to be slightly less efficient in processing time when compared to standardised, recognised protocols but was virtually imperceptible to the user. The main issue with accepting recorded ‘Unknown’ protocols was that there was no way to distinguish between correct protocols from unknown manufacturers and incorrect protocols from a scrambled recording (reflections off surfaces, dissipated through mediums, etc.). Although it was not an ideal solution, the ‘Unknown’ protocols were recorded multiple times to check for repeated codes. Scrambled codes are virtually random, and therefore any repeated codes were indicative of the real protocol pattern.

### 3.3. Characterisation of Static Gestures

**Figure 6:**
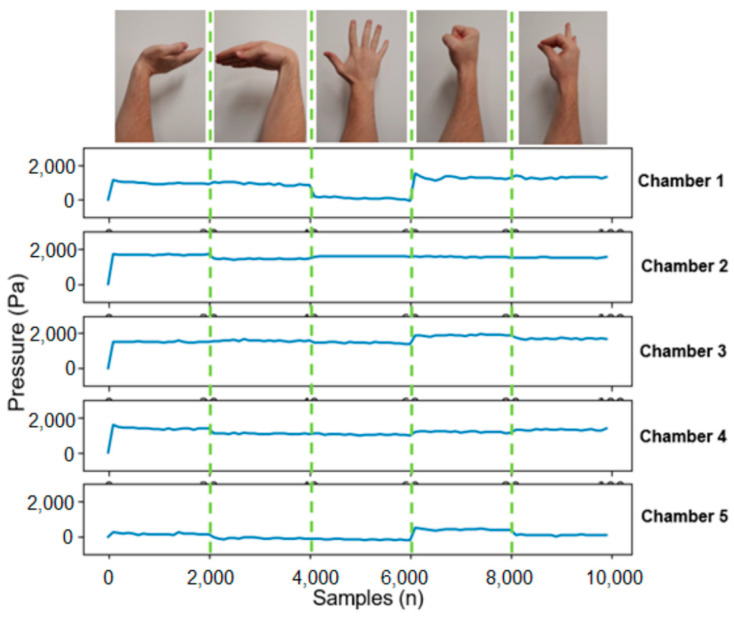
Characterisation of gesture recordings for 1 trial (2.7 s of data per gesture).

The characterisation of the static gestures (Wave Out, Wave In, Spread Fingers, Fist and Pinch) are presented in Figure 6. It was observed that the pressure output values from each of the chambers were relatively stable and showed little hysteresis effects. This partially demonstrates why filtering and rectification were not entirely necessary for this data type. The bandpass filter applied contained a function to alert the user if there was ever an abnormal spike in pressure data. The threshold was defined as a 20% change in pressure value over a period of less than a second through the duration of a single static gesture. No alerts were recorded and thus the bandpass filter was deemed not necessary for this data set but allowed for a fail-safe mechanism. 

A control armband output value was attained by placing the armband on the ground with no disturbances. This determined the base pressure already contained within each of the chambers. It was noted that the base pressure within each of the five chambers differed, but this was considered insignificant due to their small values and because the output pressures for classification are relative to initial user pressure.

Observation of the data proved very useful in identifying optimal chamber area placements to allow for highly distinguished gestures. To clarify, in Figure 6, Chamber 1 had a clear discrepancy between the Spread Fingers gesture and the Fist gesture whereas Chamber 2 had virtually no discrepancy between the gestures. To understand the reasoning behind this drastic change, it must be noted that Chamber 2 sits approximately where the pronator teres muscle lies in the forearm. The Fist and Spread Fingers gestures both do not require movement of the pronator teres and thus remain at relatively the same pressure level. In fact, Chamber 2 remains relatively the same pressure level across all 5 gestures. This does not necessarily indicate that Chamber 2 is a redundant chamber (therefore allowing for similar gesture recognition accuracies with only four chambers); however, it does indicate the lack of variety in the chosen gestures. As stated previously, these five gestures were chosen on the basis of being used extensively in the EMG-based gesture recognition literature. These results indicate that having a gesture that requires pronation of the forearm would increase the variability of Chamber 2 data and thus increase its contribution to gesture definitions.

The waveforms in Figure 6 represent data from a single volunteer and can vary significantly between different individuals. Additionally, when a volunteer doffs and dons the armband between sessions, their own waveforms can differ each time. Consequently, the armband classifier typically needs to be retrained for each recording session to achieve optimal results. In this study, the calibration training time was 12 s per gesture, which was considered an acceptable trade-off to ensure that armband orientation and placement do not significantly impact the classification process.

### 3.4. Experimental Results—Feature Selection

Our framework defined optimal features to be those that increased the inter-gesture variance of data and simultaneously decreased the intra-gesture variance of data. This meant that the data of a given gesture was repeatable and consistent within itself whilst still being effectively distinguished from other recorded gestures. Initially, a mask was used to highlight which features produced an intra-gesture variance of <10% and an inter-gesture variance of >20%. These percentage thresholds were also varied, but ultimately, this method proved to be ineffective as a feature could be deemed effective for one chamber but not another, even within the same gesture. Instead, the thresholds were removed, and features were ranked by the intra-gesture and inter-gesture variance average scores across the chambers. This whole process highlighted a potential alternative for classifier selection in future work. It may be beneficial to have a classifier model allocated to each chamber (in parallel) and then the amalgamation of the predictions is fed into an overall classifier (in series) to decide the final gesture based on constituent predictions.

Based on our ranking method, the top 50% of features were chosen as being the most useful. This included (in order) the following features: RMS, IAV, SSI, VAR, MAV1, MAV2 and MAV3. It was observed that feeding classifier models these features (thereby reducing the feature by half from 14 to 7) resulted in improvements in prediction accuracy (approximately 2–3%). This may seem insignificant, but note that this was for a pre-established effective/customised feature set and thus it demonstrated a significant improvement to standardised feature sets. In fact, the removal of the features altogether resulted in an accuracy drop between 16 and 37%, despite the relative stability in the pre-processed pressure values (it is believed this drop would be even more significant for EMG-based systems due to a relatively higher variation in the EMG signals). This drop ultimately signifies the importance of the feature selection process.

A factor that should be considered in the results obtained is that the effectiveness of these features was tested independently of each other; that is, feature combinations were not analysed. This could result in two or more features being classed as effective—independently they would increase the prediction accuracy but combined would not further increase the prediction accuracy because they are describing similar class differentiations. It is hypothesised that this may be the case for features such as MAV1, MAV2 and MAV3. The ability to distinguish effective features was additionally hindered by the difference in rankings of intra-gesture and inter-gesture variances. Some features were ranked highly in having a large inter-gesture variance (positive attribute), but simultaneously had a low-ranking score due to their large intra-gesture variance (negative attribute), e.g., TM3. When it came to these edge cases, higher scores in the inter-gesture variance rankings were weighted more than intra-gesture variance scores. It was ascertained that two gestures could be significantly varied within themselves and still be sufficiently distanced from each other for easy differentiation.

### 3.5. Experimental Results—Classifier Model Selection

#### 3.5.1. Inter-Trial and Inter-Session Gesture Recognition

The seven classifier models used in this study were evaluated in their performance with respect to accuracy, log loss and training/prediction times. Firstly, the classifier models were fed for the participant sessions individually to determine the inter-trial gesture recognition results.

The average results of all participants for the inter-trial gesture recognition capabilities are summarised in Table 1. Classifier model inter-trial quantitative results. The LDA classifier model is highlighted as the optimal model due to its accuracy score being the highest recorded on average and with the least amount of standard deviation. The training/prediction time was not the smallest recorded out of all the models, which was achieved by the GaussianNB model. It was decided, however, that accuracy takes precedence as the times are so small in nature that there is an unperceivable difference between the majority of models from the user perspective.

**Figure 7:**
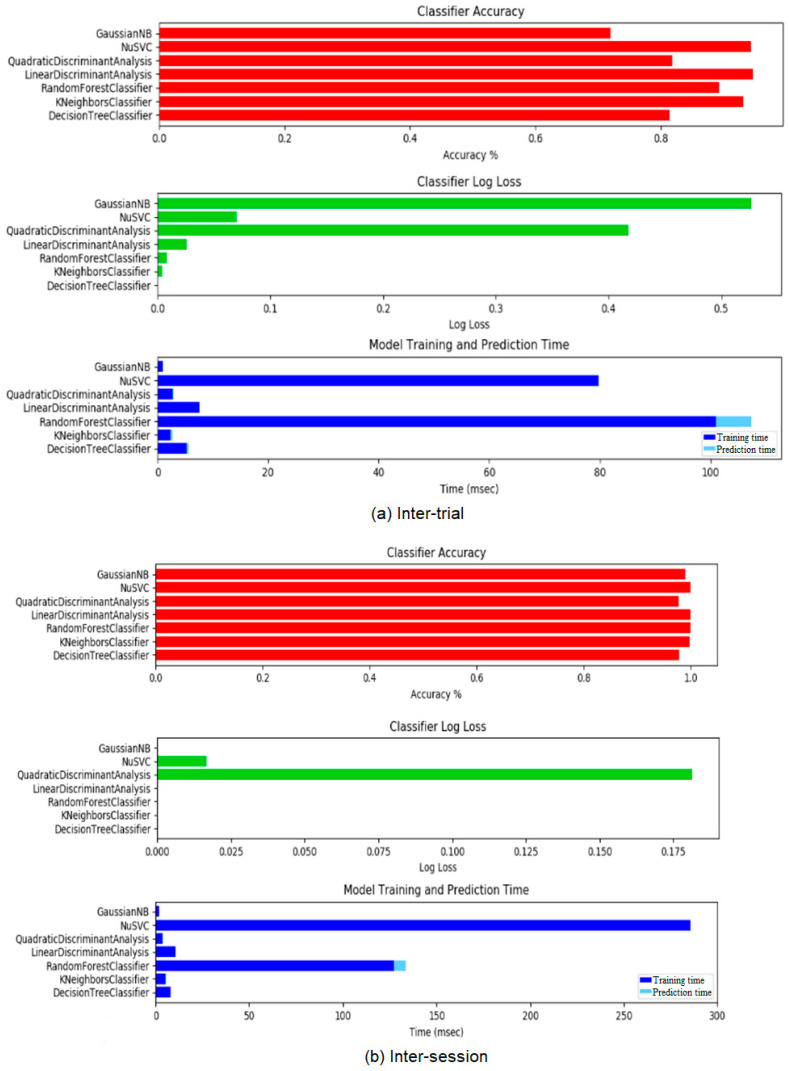
Sample of participant classifier model results with respect to accuracy, log loss and training/prediction time, note scaling ((**a**) inter-trial; (**b**) inter-session).

The average results of all participants for the inter-session gesture recognition capabilities are summarised in Table 2. The LDA classifier model was again highlighted as the optimal model due to its accuracy score being the highest recorded on average and with the least amount of standard deviation. Similar to the inter-trial gesture recognition results, the GuassianNB model was recorded to have the smallest training/prediction time but was imperceivably different from the LDA model and thus the latter was chosen as the optimal and final classifier model. A sample of the participant classifier model results with respect to accuracy, log loss and training/prediction time is presented in Figure 7.

#### 3.5.2. LDA Classifier Model Certainty/Confusion Matrices

**Figure 8:**
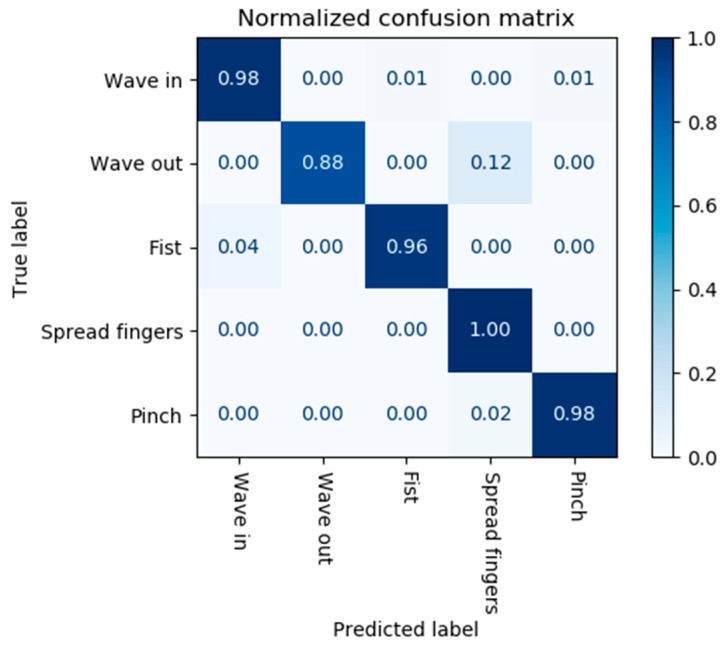
Normalized confusion matrix for given participant sample with LDA classifier model.

Following the selection of the LDA classifier model as the primary machine learning model, normalised confusion matrices (Figure 8) were generated for each participant to visualise the certainty of the predictions. Qualitative observations of these matrices highlighted some interesting conclusions that could be drawn from the data.

There were multiple cases of minor labelling confusions between the ‘Wave Out’ and ‘Spread Fingers’ gestures. It is postulated that these minor confusions may be the result of common muscle groups being displaced in the posterior forearm (e.g., extensor digitorum and extensor carpi ulnaris). Furthermore, the ‘Wave Out’ gesture could understandably be perceived as a continuous form of ‘Spread Fingers’, i.e., there is no significant change in finger/arm movements between the two. If a participant is feeling fatigued or bored, their ‘Wave Out’ gesture may become slackened to look more like a ‘Spread Fingers’. 

Another interesting feature to note is the difference in participant confusion matrices as a whole. Some participants had exceptionally high scores (97%+) across all gesture labels while those that had poor label predictions tended to over-predict a single gesture instead of an even distribution of incorrect labelling. It was noted that those with exceptional scores tended to have the armband pressed tighter against their forearm. This intuitively makes sense as pressure sensor chambers of the armband are able to be displaced with greater magnitude and be affected more by deep muscles. The tightness of the armband was not formally recorded as a quantitative or qualitative measure as part of the experimental procedure. The prototype armband used in this body of work had two circumference sizes (through the detachment of a non-sensing chamber, which does not include stretching). In an ideal scenario, the armband would be customised through 3D printing techniques to fit the individual’s forearm adequately [13].

### 3.6. Comparison with Existing Literature on Offline Gesture Recognition Results

Comparison of the original framework to the existing literature is made difficult by the fact that none of the literature has performed gesture recognition with a device working on the same principle as the pFMG armband described in this study. Comparisons are further complicated by varying parameters including differing numbers of gestures; differing data acquisition methods and devices; differing machine learning systems; differing recording times per gesture and variabilities in participant pools. The extended framework is even more difficult to compare as it incorporates quasi-dynamic gestures which is an underdeveloped area of research in the gesture recognition literature.

The hand gesture recognition performance by the proposed system was compared to some previously reported works, shown in Table 3. It was decided to set the proprietary Myo armband system (developed by Thalmic Labs [32]) as the benchmark for comparison of gesture recognition systems. Although the Myo armband is an EMG-based acquisition device, it is often used in comparison to FMG-based acquisition devices as they are used in similar applications and have somewhat similar recognition accuracies [26,27]. Furthermore, the Myo has extensively been used in academic and clinical studies [33,34,35,36]. Lastly, the five classes of gestures used in the control of the Myo armband are ‘Wave In’, ‘Wave Out’, ‘Pinch’, ‘Fist’ and ‘Spread Fingers’. As these gestures are associated with the Myo armband, they too frequently appear in gesture recognition studies (hence why these gesture classes were utilised in our framework). No such widespread standards in terms of acquisition device or gesture classes could be found for an FMG-based armband and/or framework.

The literature has reported the Myo proprietary system to have an average accuracy score of 83% [33,37]. This score was based on a dataset of EMG recordings obtained from 10 participants, but no recording times per gesture or processing times were included within these reports.

An EMG-based framework [33] claimed to achieve a greater recognition accuracy of 86% using the Myo armband and the same five gesture classes. This framework utilised k-Nearest Neighbour and dynamic time-warping algorithms as part of their machine learning model structure. Their study utilised 10 participant subjects and obtained a training set of 10 s of data per gesture. The gesture recognition system worked in real-time and was able to produce gesture predictions every 250 ms on average.

Another study used both an EMG- and FMG-based framework to determine the gesture recognition accuracy of American Sign Language (ASL) digits 0–9 [38]. Five participants performed ten trials each to assess the system. Each trial contained five seconds of data per gesture and five seconds of rest between gestures. For the EMG-based system, this work achieved a recognition accuracy of 81.5%. For the FMG-based system, a slightly reduced accuracy of 80.6% was obtained. The system had more gestures to classify compared to the previously mentioned studies, but also contained significantly more data which are factors that need to be considered when assessing the efficacy of these results.

An FMG-based study [10] claims to have achieved an accuracy of 89.4% in cross-trial evaluations for classifying 48 hand gestures. The armband used 16 Force Sensitive Resistors (FSRs) and was long enough to cover both the forearm and wrist. The hand gestures involved 16 sign language gestures, 16 individual finger movements and 16 grasps. The individual recording and processing time per gesture was not clearly specified but it was mentioned that a session consisted of performing each gesture for 5 s and resting for 5 s, repeating 10 times each. This would result in a 40 min long session and a second session was used to further increase the gesture dataset (after approximately 2 h rest).

The performance of the gesture recognition component of our framework was evaluated by taking the average accuracy from the results of 12 participant subjects. As stated in Section 3.5.1, the final classifier model used in the original framework gesture recognition system was the LDA model. To summarise the final results, the gesture recognition system was able to achieve an inter-trial accuracy score of 88.93 ± 8.70%. The inter-trial dataset consisted of approximately 6 s worth of training data and 3 s worth of testing data. The inter-session gesture recognition accuracy, based on training and testing datasets of 12 and 6 s of data per gesture, respectively, was found to achieve an accuracy score of 92.2 ± 9.35%.

It should be noted that the accuracy scores reported are for the individual gesture predictions made by the LDA classifier. In the final implementation of the system for online control of a device, post-processing gathers as many as 10 predictions per second to create a final gesture prediction that is greater in accuracy than its constituent predictions. It is hypothesised that the existing literature may also have implementations of post-processing to increase the final accuracy; however, this is not frequently reported in the literature due to its inability to be compared to others as a singular process.

**Figure 9:**
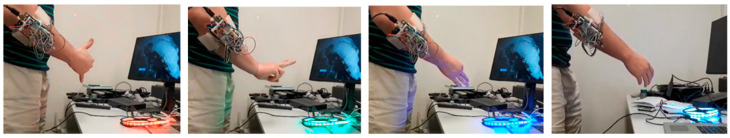
Demonstration of customised gestures for device teleoperation—gestures for shaka, peace, okay and rest (control) are depicted here.

The primary reason for using the five chosen gesture classes was for easy comparison to the existing literature. Although not quantitatively measured in this study, numerous other gestures were tested (Figure 9) to ensure that a user could effectively use their own custom gestures to control their devices.

**Table 3:** Summary of similar gesture recognition studies.

Device/Study	Type	Avg. Accuracy	Gestures	Participants	Data per Gesture	Notes
Myo Armband [32]	EMG-based	83%	Wave In, Wave Out, Pinch, Fist, Spread Fingers	10	Not specified	Extensively used as a benchmark; lacks detailed recording times and processing
EMG-based Framework [33]	EMG-based	86%	Wave In, Wave Out, Pinch, Fist, Spread Fingers	10	10 s	Utilises k-Nearest Neighbour and dynamic time-warping algorithms
ASL Digits Recognition [38]	EMG-based	81.50%	ASL Digits 0–9	5	5 s	Performed 10 trials each; uses EMG for recognition
ASL Digits Recognition [38]	FMG-based	80.60%	ASL Digits 0–9	5	5 s	Similar study using FMG for recognition
FMG-based Study [10]	FMG-based	89.40%	48 hand gestures including sign language, finger movements, and grasps	Not specified	5 s	Uses 16 FSRs; covers forearm and wrist; 10 data collections per 40 min session, session repeated
**Our study (inter-trial)**	pFMG-based	88.93%	Wave In, Wave Out, Pinch, Fist, Spread Fingers	12	6 s	LDA model, post-processing of 10 predictions made per second
**Our study (inter-session)**	pFMG-based	92.2%	Wave In, Wave Out, Pinch, Fist, Spread Fingers	12	12 s	Armband not adjusted between sessions.

### 3.7. Experimental Results—Online IR Device Teleoperation

The online IR device teleoperation results were limited only by the gesture recognition accuracy of the classifier and by a participant’s ability to correctly point at the activating device. The prototype armband incorporated the IR transmitter onto the top of the armband placed on the forearm and thus the user was required to physically point their arm whilst activating a gesture. This partially limited gestures to the longitudinal axis. A real application may benefit from using IR boosters/extenders to allow more freedom of rotational movement and/or incorporating a more powerful IR transmitter with increased dispersive capabilities.

**Figure 10:**
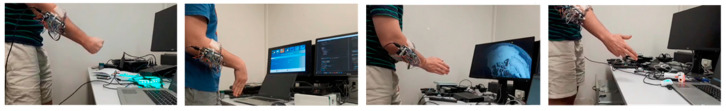
Operation of an LED strip, TV set box, movie hard drive player and laser tag equipment, from left to right. Also demonstrates some more custom gestures that are intuitive to the application (e.g., pistol gesture for shooting mechanic in laser tag game).

A variety of devices were able to be activated throughout the duration of this study, demonstrating an ability to emulate a range of functionalities. These devices (shown in Figure 10) included televisions, media systems, temperature units, entertainment/gaming systems and utilised manufacturer code types. Although incorporating compatibilities for 17 unique protocol types, it was observed that the majority of devices fell under only a few types, the primary one being the NEC protocol.

### 3.8. Experimental Results—Extended Framework

**Figure 11:**
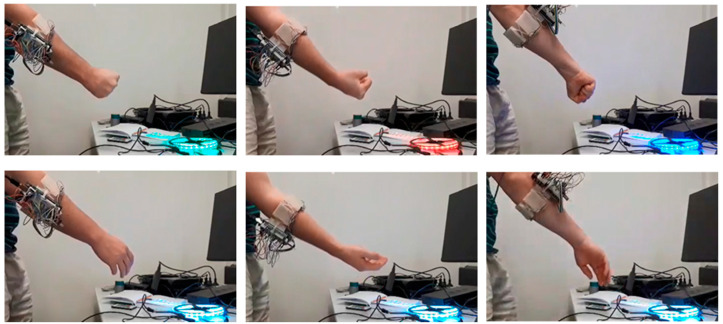
Spatial augmentation demonstration—**Top row**: Fist gesture augmented by arm orientation changes LED light colour. **Bottom row**: Without activating a gesture, the orientation does nothing.

Spatial augmentation of the system was deemed to be sufficient for the short-term goal of attaining quasi-dynamic gestures. Users were able to perform a static gesture and then extend the functionality of that gesture by modifying their arm orientation. As demonstrated in Figure 11, a ‘Fist’ gesture was able to turn an LED strip on and, with longitudinal orientation of the arm, modify the LED colours during the post-prediction phase. Lateral and medial rotation of the arm changed the LED strip to red and blue, respectively. Figure 11 also demonstrates how this augmented functionality is a post-prediction process, that is, if a gesture is not identified the device is not “locked on” and therefore orientation does nothing.

Figure 11 showcases a discrete implementation of the spatial augmentation capabilities, that is, the LED strips can be in the colour state of green, red or blue with no intermediate colours between states. A continuous gradient of colour change was deemed inefficient using quasi-dynamic gestures alone. The implemented proportional control system allowed for a more continuous variable that was proportionate to the difference between the orientated gesture position and the initial gesture position. This variable produced a spectrum of possible boundaries, the resolution determined by the user’s desire. A DVD player’s fast-forwarding functionalities were tested using proportional control. The more a ‘Wave Out’ gesture was laterally rotated, the faster the fast-forwarding function would operate. An issue that arose from this, however, was that the user’s hand may initially be facing the device, but varying the orientation affected the IR transmitter’s line of sight to the device. Whilst IR communication can generally be achieved across a wide field of view at short distances, it may be preferable to limit quasi-dynamic gestures to rolling motions (as opposed to pitch or yaw). Similar to the original framework, possible solutions beyond the prototype armband used would be to incorporate IR extenders/boosters, move the IR transmitter to somewhere more practical on/in the body/environment or utilise a more powerful IR transmitter with a greater field of view.

### 3.9. Final Remarks on Experimental Results

The key point to note from the results gathered and its subsequent comparison with existing results in the literature is that our designed framework either meets or exceeds the recognition capabilities deemed acceptable in the literature. Our armband uses fewer sensors (five) than most of the frameworks analysed in the literature. The number of gestures tested in this study was sometimes significantly lower than the research work that was designed for sign language capabilities. As no more than five gestures were tested at a time, it is presently unknown what our gesture recognition scores would be for a sign language application. For our system, it is deemed sufficient that the most popular remote-control functionalities can be served by simply a handful of gestures. Those works that incorporated more gestures, however, also tended to have significantly larger datasets for training and testing. One of the main features of our system is the turnaround time between users picking up the armband for the first time and ultimately controlling all their household devices. This can be carried out effectively within a matter of minutes—this time can be shortened even further if the user has previously saved their gesture data.

It should also be noted that the number and selection of gestures were limited to allow for easy comparison to EMG-based gesture recognition systems. Lessons learned from the testing and results of the original framework highlighted a need for better-defined static gestures. Traditionally, FMG has been compared to EMG due to the similarity in data acquisition (reading muscular signals) and its potential applications. As a result, gesture recognition methods and evaluation parameters employed for FMG have historically been guided by the standards tailored to EMG applications. It is recognised that FMG presents a substantially different signal profile to EMG, and thus it would be beneficial to explore gestures that are uniquely beneficial to FMG applications.

## 4. Conclusions

Both IR remote emulation and FMG-based gesture recognition systems were possible and existed in the industry/literature. What was lacking, however, was a demonstration of the amalgamation of these two domains. To the best of the authors’ knowledge, no other studies have detailed or implemented an FMG-based gesture recognition system for the teleoperation of consumer IR devices. This study has described and built the foundations of designing, developing and implementing such a system as a safe and wearable human–machine interface. 

This study has employed a novel pFMG armband to acquire FMG data from the upper forearm of users. This, in combination with off-the-shelf components for infrared emulation, has established a low-cost, customisable and effective acquisition device for user intent and device communication. Each of the phases of the system architecture is detailed and an in-depth analysis is provided as to the reasoning and justification for each process followed throughout the duration of this work. A particular emphasis was placed on creating a solution that was modular, adapted to the individual user and sufficiently fast to allow for ‘plug and play’ capabilities.

Two implementations of the gesture recognition system were described in this study—the original framework and the extended framework. The original framework used five static gestures that were prevalent in myography-based gesture recognition to demonstrate the effectiveness of our proposed method. The extended framework provided additional functionalities to the gestures by spatially augmenting the data to create quasi-dynamic gestures and allow for proportional control. The efficacy and performance of the frameworks were evaluated by the pFMG data obtained from 12 and 3 participant subjects for the original and extended frameworks, respectively.

In summary of the results, the optimal features for use in the gesture recognition system were found to be RMS, IAV, SSI, VAR, MAV1, MAV2 and MAV3. The optimal classifier model for both inter-trial and inter-session accuracy was found to be the LDA model. For a dataset containing 6 s worth of pFMG data per gesture, our recognition system was able to achieve an inter-trial accuracy score of 88.93 ± 8.70%. For a dataset containing 12 s worth of pFMG data per gesture, our recognition system was able to achieve an inter-session accuracy score of 92.20 ± 9.35%. Teleoperation of consumer IR devices was demonstrated through successful control of a variety of consumer devices, utilising a range of protocol modulation methods and manufacturer code types. All gestures used in the original framework were able to be augmented with spatial data and with proportional control, providing extended capabilities when teleoperating consumer IR devices.

When compared to the existing literature incorporating FMG- or EMG-based gesture recognition systems with similar gesture classes, our work has proven itself to meet or even exceed results with respect to recognition accuracy and prediction speed. It should be noted that even better accuracy scores are achieved when incorporating post-processing functionalities. Aside from recognition accuracies, the implemented system has the benefit of being very customisable, affordable, lightweight, 3D printed, impervious to sweat and does not require direct skin contact.

## Figures and Tables

**Table 1:** Classifier model inter-trial quantitative results.

Classifier Model	Accuracy (%)	Log Loss	Training/Prediction Time (ms)
GaussianNB	77.18 ± 19.95	0.068 ± 0.124	3.9 ± 1.4
NuSVC	85.32 ± 11.02	0.070 ± 0.100	72.6 ± 4.0
QDA	84.11 ± 10.25	0.226 ± 0.333	5.6 ± 1.5
**LDA**	**88.93 ± 8.70**	**0.009 ± 0.015**	**8.8 ± 1.2**
RF	83.88 ± 8.87	0.004 ± 0.005	98.6 ± 2.1
K-NN	86.43 ± 11.85	0.003 ± 0.005	5.2 ± 0.9
DT	82.14 ± 9.66	0.000 ± 0.000	5.9 ± 1.3

**Table 2:** Classifier model inter-sessional quantitative results.

Classifier Model	Accuracy (%)	Log Loss	Training/Prediction Time (ms)
GaussianNB	87.75 ± 13.62	0.128 ± 0.097	4.6 ± 0.7
NuSVC	90.68 ± 11.31	0.013 ± 0.009	273.9 ± 3.4
QDA	90.12 ± 11.84	0.105 ± 0.096	6.8 ± 1.2
**LDA**	**92.20 ± 9.35**	**0.010 ± 0.009**	**16.1 ± 1.1**
RF	88.15 ± 10.57	0.001 ± 0.001	161.3 ± 4.2
K-NN	91.20 ± 11.90	0.001 ± 0.001	7.5 ± 0.9
DT	86.18 ± 11.19	0.000 ± 0.000	15.9 ± 2.3

## Data Availability

The data is unavailable due to privacy or ethical restrictions.
